# DNAzyme‐Based Nanostructures for Dynamic Cell Regulation and Sensing

**DOI:** 10.1002/open.202600004

**Published:** 2026-03-25

**Authors:** Haoyu Fan, Xichen Du, Ruocan Qian

**Affiliations:** ^1^ International Elite Engineering School East China University of Science and Technology Shanghai China; ^2^ Key Laboratory for Advanced Materials Feringa Nobel Prize Scientist Joint Research Center Joint International Laboratory for Precision Chemistry Frontiers Science Center for Materiobiology & Dynamic Chemistry School of Chemistry and Molecular Engineering East China University of Science and Technology Shanghai P. R. China

**Keywords:** bioimaging, biosensing, cell regulation, DNAzyme, nanostructure

## Abstract

This review systematically explores the cutting‐edge advancements in DNAzyme‐based nanostructures for dynamic cellular regulation and biosensing. As catalytic DNA molecules, DNAzymes exhibit high stability, programmability, and versatile functionality, making them powerful tools for diverse applications, including metal ion detection, controlled cell–cell interactions, and high‐resolution bioimaging. By integrating with nanomaterials such as liposomes, gold nanoparticles, and metal‐organic frameworks, DNAzyme systems have overcome inherent limitations such as nuclease susceptibility and poor cellular uptake, thereby significantly enhancing their performance and stability in complex biological environments. Key innovations discussed include logic‐gated systems for programmable cell assembly, rRNA‐activated sensors for subcellular imaging, and DNAzyme‐driven nanodevices like walkers and tweezers for amplified sensing and intracellular manipulation. These developments pave the way for novel therapeutic strategies in targeted gene silencing and combination cancer therapy. Finally, we highlight future directions focusing on context‐responsive nanocarriers, multimodal theranostic platforms, and scalable biocompatible designs to promote the clinical translation of DNAzyme technologies.

## Graphical abstract

This review explores DNAzyme‐based nanostructures for dynamic cell regulation and sensing. By merging catalytic DNA with nanomaterials, they enable precise metal‐ion detection, programmable cell–cell interactions, and high‐resolution bioimaging. These advances open new routes to smart theranostic platforms and clinical translation in precision medicine.

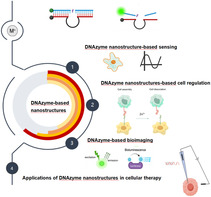

## 1 Introduction

DNAzyme is a class of catalytic DNA molecules first identified in 1994 by American scientists Ronald R. Breaker and Gerald F. Joyce through the in vitro selection methodology known as SELEX (Systematic Evolution of Ligands by Exponential enrichment). Their work demonstrated that DNA sequences could be evolved from a large random‐sequence library through iterative rounds of selection for catalytic function and amplification to specifically cleaving RNA in the presence of cofactors such as Pb^2+^, leading to the designation “deoxyribozymes” or DNAzymes. The SELEX methodology, involving iterative selection and amplification, provides a powerful and general approach for discovering functional nucleic acids (such as aptamers and DNAzymes) from combinatorial libraries, laying a crucial foundation for the development of tools in synthetic biology and molecular sensing. The paradigm that all enzymes are proteins had already been decisively overturned more than a decade earlier by the independent discoveries of ribozymes by Thomas Cech and Sidney Altman in the early 1980s. The subsequent identification of DNAzymes further expanded the realm of biocatalysis beyond proteins and catalytic RNAs (ribozymes) [1, 2, 3, 4, 5]. Together with ribozymes (RNAzymes), DNAzymes have expanded the scope of enzymology and promoted the development of diverse biocatalytic molecules.

As of 2025, a diverse array of DNAzymes has been discovered, showcasing the catalytic versatility of DNA. These enzymes are typically classified based on their core catalytic functions. Major functional categories include: RNA‐cleaving DNAzymes (e.g., the classic 10–23 and 8–17 motifs), which catalyze the site‐specific hydrolysis of RNA phosphodiester bonds. DNA‐ligating DNAzymes, which catalyze the formation of phosphodiester bonds between DNA strands. DNAzymes catalyzing other chemistries, such as DNA phosphorylation, capping, and thymine dimer repair, demonstrate the expanding catalytic repertoire of nucleic acids. The activity of virtually all DNAzymes is dependent on specific metal ion cofactors (e.g., Mg^2+^, Zn^2+^, Pb^2+^, Cu^2+^), which play crucial roles in substrate binding, transition‐state stabilization, and acid–base catalysis. Therefore, metal‐ion dependence is a fundamental and pervasive mechanistic feature across different functional classes, rather than a separate or independent classification criterion [6, 7, 8, 9]. The field of DNAzyme research is propelled by two complementary, yet distinct objectives. The first is a fundamental inquiry into the catalytic potential of DNA. This pursuit has led to the in vitro evolution of DNAzymes capable of a broad spectrum of chemical transformations, extending far beyond RNA cleavage to include DNA ligation, kinase‐like phosphorylation, 5′ capping, and even thymine dimer photorepair. These achievements are motivated by questions in basic enzymology and the origins of life, showcasing the intrinsic catalytic breadth of nucleic acids. The second objective is the development of DNAzymes as programmable molecular tools. Here, their attributes‐high specificity, catalytic activity, and designability‐are harnessed for precise molecular regulation, which we define as the ability to detect, image, or catalytically alter a target molecule within a complex system. A major focus of this applied research is its translation into biomedical applications, a term encompassing distinct endeavors such as therapeutic intervention (e.g., gene silencing), diagnostic sensing, and research‐focused intracellular imaging and manipulation [10, 11, 12, 13, 14, 15]. In recent years, by combining DNAzymes with nanomaterials (e.g., liposomes and gold nanoparticles (AuNPs)), their stability, targeting ability, and delivery efficiency have been significantly improved, greatly advancing innovative applications in biomedicine and nanotechnology [16, 17, 18].

Structurally, many well‐studied DNAzymes, particularly the canonical RNA‐cleaving types such as 10–23 and 8–17, are composed of a catalytic core flanked by substrate‐recognition arms that bind complementary RNA targets via Watson–Crick base pairing. The catalytic core often requires specific metal ions (e.g., Mg^2+^, Zn^2+^) as cofactors to mediate reactions such as RNA cleavage. It is important to note that structural motifs can vary significantly for DNAzymes catalyzing other types of reactions (e.g., ligation, phosphorylation), which may not adhere to this simple bipartite “core‐arms” architecture. Although DNAzymes exhibit attractive properties for molecular regulation, their translation into biomedical applications faces several challenges. Many of these barriers are shared with other nucleic acid‐based therapeutics and probes, but they manifest in specific ways for DNAzymes: susceptibility to degradation by nucleases, resulting in a short in vivo half‐life; inefficient cellular delivery due to electrostatic repulsion and lack of inherent targeting; potential conformational instability or inactivation in complex physiological environments; and nonspecific adsorption to proteins, which can reduce bioavailability and signal‐to‐noise ratio. Furthermore, their catalytic activity is often highly dependent on local cofactor availability (e.g., specific metal ions) and can be sensitive to fluctuations in pH, adding another layer of environmental constraint within cells [19, 20, 21].

Nanotechnology provides tailored strategies to overcome the inherent limitations of DNAzymes, with material design often dictated by the specific biological context and intended application (e.g., in vitro detection, intracellular sensing, or in vivo therapy). These strategies can be categorized based on their primary mechanistic role: (1) Enhancing Stability and Circulation: To combat nuclease degradation and extend in vivo half‐life, DNAzymes can be encapsulated within protective nanostructures that create a physical barrier. Examples include liposomes, polymeric NPs, and metal‐organic frameworks (MOFs), which have been shown to significantly prolong stability in biological fluids [22]. (2) Facilitating Cellular Delivery and Targeting: Overcoming the electrostatic repulsion with cell membranes and achieving cell‐specific localization are critical for intracellular applications. This is addressed through two complementary approaches: Enhanced Uptake: Nanocarriers like AuNPs or mesoporous silica, especially when modified with cell‐penetrating peptides, promote efficient cellular internalization via endocytosis. Active Targeting: Functionalization of nanocarriers with targeting ligands (e.g., antibodies, aptamers, folic acid) enables precise accumulation at disease sites. For instance, aptamer‐mediated DNAzyme nanoassemblies allow for specific recognition and regulation of cancer cells [23]. (3) Enabling Context‐Responsive Activation and Signal Control: To minimize off‐target effects and improve signal fidelity in complex environments, nanomaterials can be engineered for conditional activity. Stabilization/Activation: Certain nanomaterials can shield the DNAzyme from interfering factors or enable controlled release in response to specific microenvironmental cues (e.g., pH, enzymes, or redox conditions). Signal Amplification & Noise Reduction: Integration with signal transduction modalities is crucial for sensitive detection. Nanomaterials facilitate this through mechanisms such as fluorescence quenching/recovery (e.g., using graphene oxide) [24] or plasmonic enhancement, significantly improving the signal‐to‐noise ratio and detection limits. Crossing Biological Barriers: For therapeutic applications targeting privileged sites like the central nervous system, biomimetic nanocarriers (e.g., engineered liposomes, exosomes) can be designed to assist DNAzymes in traversing formidable biological barriers such as the blood‐brain barrier [25]. This principled integration of nanomaterials transforms DNAzymes from vulnerable molecular entities into robust, context‐aware “nanodevices,” paving the way for their reliable use in complex biological settings.

This review systematically summarizes recent advances in DNAzyme‐based nanodevices for sensing, cellular imaging, and gene therapy, aiming to provide theoretical insights and technical prospects for future translational research.

## 2 DNAzyme Nanostructure‐Based Sensing

**FIGURE 1:**
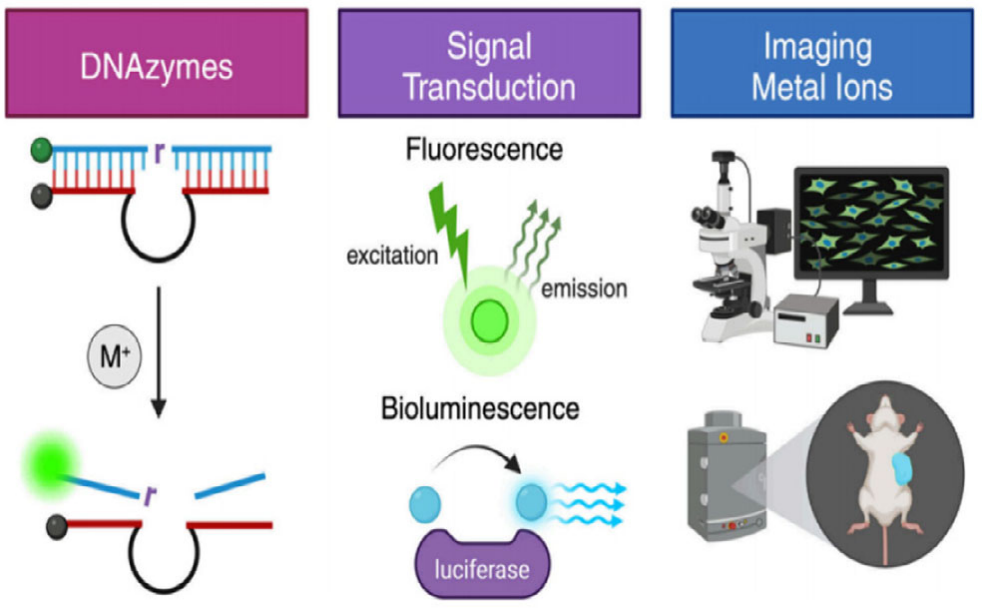
Schematic Illustration of DNAzyme Signal Transduction Mechanisms. Reproduced with permission. [Ref. 33] Copyright 2025, American Chemical Society Publications.

DNAzyme‐based sensing is one promising direction in which significant research efforts have been invested. Unlike conventional techniques like gas chromatography‐mass spectrometry(GC‐MS), high‐performance liquid chromatography‐mass spectrometry (HPLC‐MS), polymerase chain reaction (PCR), plate counting, and enzyme‐linked immunosorbent assays (ELISA), etc. In contrast, DNAzyme‐based sensing offers a novel alternative [26, 27, 28, 29, 30]. DNAzyme‐based sensors are especially remarkable for their excellent specificity, allowing the detection of very ions at trace amounts. Their inherent programmability and ease of integration into other platforms make them ideal for complicated biological environments. When coupled with techniques such as fluorescence and magnetic resonance imaging [31, 32], it provides researchers with a variety of alternatives and enhances the precision of monitoring biological processes and improves treatment outcomes and diagnosis, as shown in Figure 1 [33].

### 2.1 Comparison of DNAzyme‐Based Sensors With Other Sensor Types

The selection of an optimal biosensing platform depends on a balanced consideration of key performance parameters, including sensitivity, specificity, response time, stability, cost, and technological maturity. Here, we systematically compare DNAzyme‐based sensors with three well‐established counterparts: general chemical sensors, electrochemical sensors, and protein‐based biosensors. A quantitative summary of their representative performance metrics is provided in Table 1.

**TABLE 1:** Comparative analysis of key performance parameters for various biosensing platforms.

Sensing platform	Typical limit of detection (LOD)	Response time	Selectivity/specificity	Key advantages	Major limitations	Representative applications
DNAzyme‐based sensors	pM‐nM range (e.g., Zn^2+^: ~0.2 nM)	Minutes to hours	High (sequence‐specific recognition and catalysis)	High specificity and programmability; good chemical stability; suitable for intracellular imaging.	Susceptible to nuclease degradation; low cellular delivery efficiency; activity often buffer‐dependent.	Metal ion detection; intracellular RNA imaging; programmable molecular circuits.
Electrochemical sensors	nM‐µM range	Seconds to minutes	Moderate (prone to matrix interference)	Rapid response; easy miniaturization and integration; low cost; suitable for point‐of‐care testing.	Relatively poor selectivity; electrode fouling; stability issues with biorecognition elements.	Food safety screening; portable monitoring of physiological markers (e.g., glucose).
Protein‐based sensors (e.g., ELISA)	pM‐nM range	Several hours	Very High (antibody‐antigen recognition)	Extremely high specificity; mature and automated technology; well‐established for clinical use.	Time‐consuming; requires labeling; complex procedure; unsuitable for real‐time in vivo monitoring.	In vitro diagnosis of disease biomarkers; protein quantification.
PCR‐based assays	aM‐fM range (for nucleic acids)	1–2 h	Exceptionally High (sequence amplification)	Ultra‐high sensitivity; gold standard for nucleic acid detection.	Requires thermal cyclers; prone to contamination; not applicable for non‐nucleic acid targets.	Pathogen nucleic acid testing; gene expression analysis.
Fluorescent protein sensors	nM‐µM range (intracellular)	Milliseconds to seconds	Moderate to High	Ideal for real‐time dynamic imaging in live cells; genetically encodable; high spatiotemporal resolution.	Prone to photobleaching; background fluorescence; complex design and optimization.	Dynamic imaging of ion concentrations in cells; protein–protein interaction studies.

DNAzyme‐Based Sensors occupy a unique niche, merging the programmability and design flexibility of nucleic acids with catalytic signal amplification. They achieve exceptional specificity and can be engineered for complex logic operations, making them powerful for in vitro detection and intracellular sensing of specific targets like metal ions or RNA. Their main challenges are susceptibility to nuclease degradation, dependence on cofactors, and current hurdles in large‐scale manufacturing [26, 27, 28, 29, 30, 31, 32, 33].

General chemical sensors excel in practicality, offering rapid response, low cost, and ease of integration into portable or wearable devices for continuous, real‐time monitoring (e.g., of biomarkers in bodily fluids). However, they often suffer from insufficient selectivity and susceptibility to environmental interference, limiting reliability in complex matrices [34, 35, 36, 37].

Electrochemical sensors translate chemical interactions into measurable electrical signals, achieving high sensitivity—especially when enhanced with nanomaterials—and are ideal for miniaturized, on‐site detection. Their primary drawbacks include relatively poorer selectivity, electrode fouling, and dependence on the stability of biological recognition elements [38, 39, 40, 41]. While potentially lagging behind DNAzyme sensors in ultimate molecular specificity, they surpass them in technological maturity and ease of system integration.

Protein‐based biosensors (e.g., enzyme or antibody‐based) leverage exquisite natural specificity and, for enzymes, inherent catalytic signal amplification. They are unparalleled for dynamic imaging and analysis within native physiological environments. Their limitations stem from the intrinsic fragility of proteins: poor stability, potential immunogenicity, and complex, costly production [42, 43, 44].

In summary, the choice among these platforms involves clear trade‐offs. Chemical and electrochemical sensors are the champions of field‐deployable, rapid, and cost‐effective monitoring. Protein‐based sensors are the gold standard for fidelity and function within complex biological systems. DNAzyme sensors offer a powerful synthetic biology tool for highly specific, programmable detection, particularly where conventional bioreceptors are unavailable or insufficient. The future lies in creating hybrid systems, such as integrating DNAzyme's precise recognition with electrochemical transducers’ rugged output, to overcome individual limitations and unlock new sensing capabilities.

### 2.2 DNAzyme Nanostructure‐Based Cell Sensing

DNAzyme‐based nanostructures demonstrate significant application prospects in the field of cellular sensing. By integrating the catalytic function of DNAzymes with the programmable properties of nanomaterials, these structures enable highly sensitive and spatiotemporally resolved detection of intracellular targets. These systems not only possess excellent molecular recognition capabilities but also enable signal amplification and multiplexed responses, providing novel analytical tools for life science research and medical diagnostics [45, 46, 47].

Rigid scaffolds, exemplified by tetrahedral DNA nanostructures (TDNs) or cube‐based frameworks, allow precise assembly of DNAzyme molecules. These architectures not only promote efficient cellular internalization but also protect DNAzymes from nuclease degradation, thereby enhancing their stability in intracellular environments [48]. Functional nanocomposite systems further expand their application potential: AuNPs leverage surface plasmon resonance effects and efficient fluorescence quenching properties to construct “off‐on” fluorescent sensors [49]; MOFs enable high‐efficiency loading and controlled release of DNAzymes and reporter molecules [50]; magnetic NPs facilitate targeted delivery and separation enrichment under external magnetic guidance [51].

Moreover, dynamic self‐assembly systems employ allosteric DNAzyme designs that undergo conformational changes in the presence of targets such as metal ions or mRNA, thereby triggering cascade hybridization chain reactions or activating DNA nanomachines to achieve signal amplification and real‐time imaging [52].

In cellular sensing applications, DNAzyme nanostructures can be utilized for metal ion imaging—leveraging their high selectivity for ions such as Zn^2+^, Cu^2+^, and Pb^2+^ to enable fluorescence or Raman imaging of dynamic distributions within live cells, and even distinguish ion concentrations across subcellular compartments [53]. For nucleic acid biomarker detection, these systems can catalyze the cleavage of tumor‐related genes such as miR‐21, Survivin, or c‐Jun and activate reporter genes. When integrated with CRISPR/Cas systems or exosome delivery strategies, they further enhance detection sensitivity and tissue penetration capabilities [54]. Additionally, the DNAzyme‐delivered coordination nanosystem (ZDD) achieves precise targeting of tumor endogenous iron metabolism through a synergistic DNAzyme delivery and Fe^3+^‐responsive mechanism, simultaneously inducing GPX4‐dependent ferroptosis while blocking the VEGFR2‐mediated angiogenesis pathway, thereby enabling efficient and low‐toxicity tumor therapy [55].

### 2.3 DNAzyme Nanostructure‐Based Metal Ion Sensing

#### 2.3.1 Mechanism of DNAzyme‐Based Metal Ion Sensing

**FIGURE 2:**
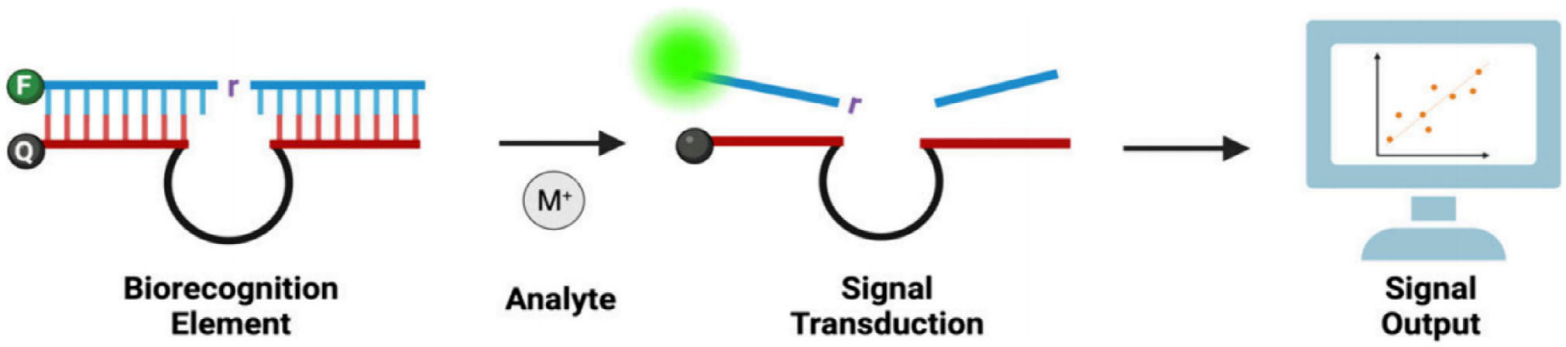
Overview of DNAzyme‐Based Signal Transduction Mechanism. The analyte (gray) specifically recognizes and binds to the DNAzyme bioreceptor (red). A signal transduction unit (green), composed of a fluorophore and a quencher, detects this molecular interaction and converts it into a raw detection signal. This signal is processed by an electronic system and ultimately converted into a visual output (light blue). This process, referred to as signal transduction, functionally couples the biorecognition event with the signal output event. Reproduced with permission. [Ref. 33] Copyright 2025, American Chemical Society Publications.

The sensing mechanism for metal ions based on DNAzymes is relatively straightforward in principle (Figure 2) [33]. The process initiates when the metal ion binds to the specific recognition sequence of the DNAzyme. These recognition sequences contain specialized binding arms that are connected to the substrate strand at one end and anchored via specific secondary structures to the catalytic core region.Upon coordination with the metal ion, the DNAzyme undergoes a conformational change that induces cleavage of the substrate strand—a step that is critical for biosensing as it triggers subsequent signal transduction events. Moreover, most DNAzymes are capable of catalyzing site‐specific cleavage of phosphodiester bonds. This catalytic activity is triggered by the metal ion‐induced conformational change that activates the catalytic core. The activated DNAzyme then cleaves its complementary RNA or DNA substrate, leading to a change in the melting temperature of the system and resulting in the dissociation and release of the substrate strand from the enzyme strand. Finally, by conjugating signal transduction elements (such as fluorophores, chromophores, or chemiluminescent groups) to either the enzyme or substrate strand, a detectable physical signal‐such as fluorescence emission, color change, or chemiluminescence‐can be generated upon cleavage, effectively indicating the presence of the target metal ion. These signals can be further quantitatively analyzed to determine the specific concentration of the metal ion. To enhance detection sensitivity and signal intensity, various amplification strategies can be employed, such as multi‐round cycles of binding‐cleavage‐signal generation to achieve signal amplification [33, 56, 57].

#### 2.3.2 Nanostructures in DNAzyme‐Based Metal Ion Detection: Roles and Value

DNAzyme‐based metal ion sensors have been profoundly enhanced by the integration of functional nanostructures, which play multifaceted roles that significantly elevate sensor performance across various practical scenarios. These nanomaterials—including AuNPs, DNA TDNs, MOFs, and magnetic NPs‐serve as tunable scaffolds that not only offer high‐density and oriented immobilization of DNAzyme probes, but also greatly enhance their operational stability under complex chemical and biological conditions. Through mechanisms such as plasmonic enhancement, fluorescence quenching, and electrocatalytic amplification, nanostructures considerably lower detection limits, frequently reaching picomolar or even femtomolar levels, while also enabling multimodal signal readouts spanning fluorescent, electrochemical, colorimetric, and surface‐enhanced Raman scattering (SERS) techniques [58, 59, 60, 61].

Furthermore, the functionalization of these nanostructures facilitates efficient cellular internalization and targeted delivery, allowing for real‐time imaging and quantification of metal ions such as Zn^2+^, Cu^2+^, and Pb^2+^ within living cells and subcellular compartments. Such capabilities are indispensable for advancing studies in metal ion metabolism, neurobiology, and toxicology. The application scope of these hybrid sensors extends from environmental water quality monitoring and food safety assurance—enabling trace detection of heavy metal contaminants—to biomedical diagnostics and personalized medicine, where they contribute to noninvasive disease marker monitoring and therapeutic effect evaluation [62, 63, 64, 65, 66]. Despite these promising features, challenges remain in achieving large‐scale manufacturability, ensuring long‐term stability in real‐world environments, and standardizing performance across diverse sample matrices. Future developments are expected to focus on “smart” stimuli‐responsive nanostructures, streamlined integration with portable readout devices, and the incorporation of machine learning tools for data interpretation, thereby broadening the impact and accessibility of DNAzyme‐nanostructure platforms in both field‐deployable and clinical settings [67].

## 3 DNAzyme Nanostructures‐Based Cell Regulation

### 3.1 Metal Ion Mediated Control of Cell–Cell Interactions

The use of RNA‐cleaving DNAzymes to control cell–cell interactions represents an intelligent and versatile approach. It involves installing artificial receptors on cell membranes to mediate specific interaction and communication between cells [68, 69]. DNAzyme can be fixed on lipid bilayers via lipid anchors or sent into cell by nanopipette.Our group has conducted extensive research in this area [70]. For instance, we have managed to realize metal ion mediated control of cell–cell interaction.Through anchoring DNAzyme on lipid bilayers, they formulated “and” and “or” mechanisms for cancer cell and T‐cell to be bound and disassociated and therefore facilitating therapies for cancers [71].

### 3.2 DNAzyme‐Based Cell–Cell Interaction Control System

**FIGURE 3:**
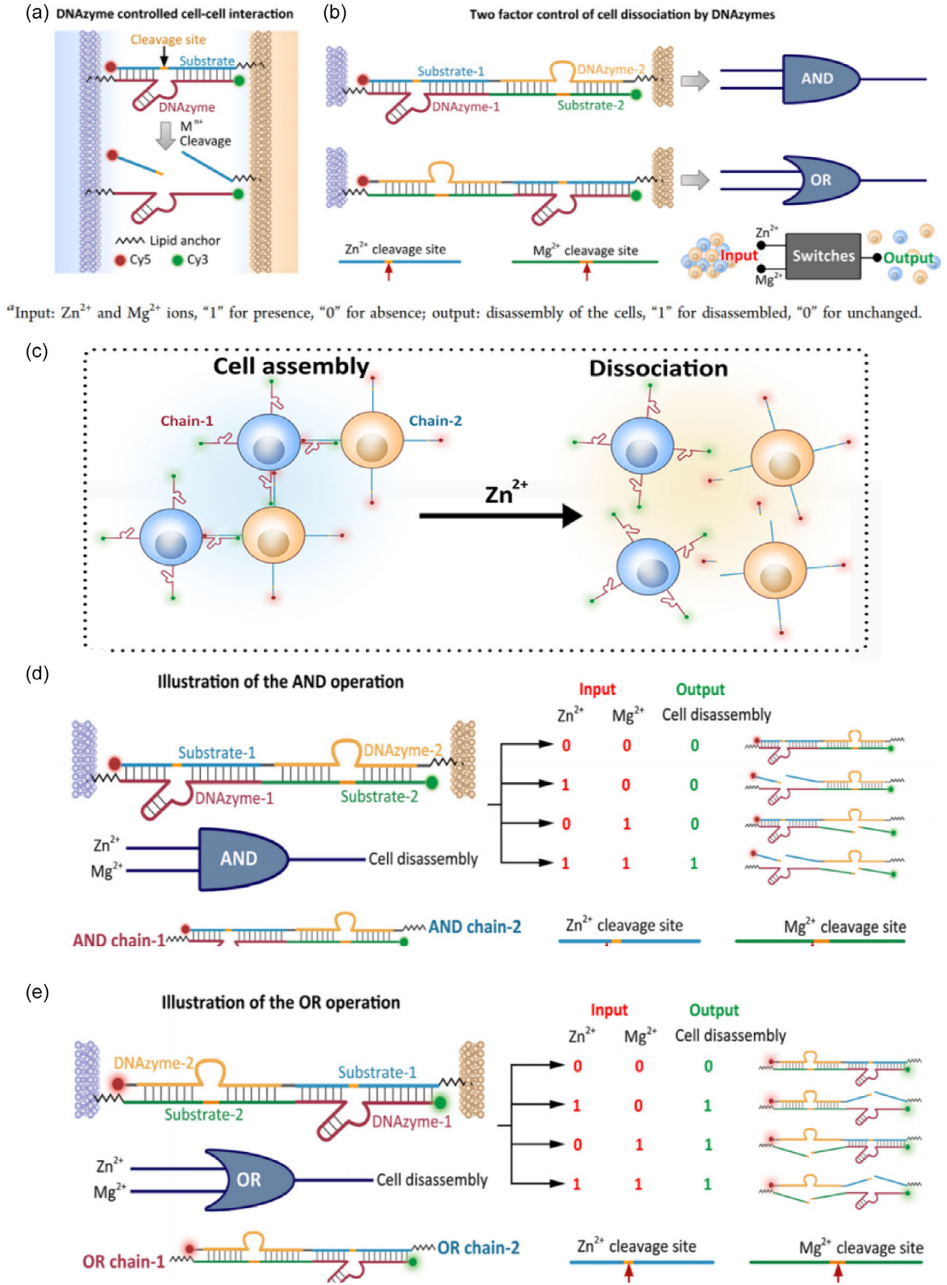
DNAzyme‐Mediated Intercellular Interaction Regulatory System. (a) After cells modified with DNAzyme and substrate are embedded into the cell membrane, controllable cell–cell assembly is achieved through specific hybridization between the DNAzyme and its substrate strand. (b) A dual‐chain molecular switch structure constructed using Zn^2+^‐and Mg^2+^‐specific DNAzymes enables dual‐input‐controlled disassembly of cell assemblies via “AND” and “OR” logic gate operations. This system connects discrete cells through DNA hybridization, forming macroscopic cell assembly structures. The disassembly behavior can be coordinately regulated by the molecular switch logic and the two input signals (Zn^2+^and Mg^2+^), achieving programmable dynamic response outputs. (c) A Zn^2+^‐specific RNA‐cleaving DNAzyme (Chain‐1) and its corresponding substrate strand (Chain‐2) were employed as a molecular regulatory switch to achieve programmable assembly and disassembly of HeLa cells. (d) AND logic gate function: The cell assembly disassembles only in the simultaneous presence of both Zn^2+^and Mg^2+^. (e) OR logic gate function: The presence of either Zn^2+^ or Mg^2+^ alone is sufficient to induce disassembly of the cell assembly. Reproduced with permission. [Ref. 71] Copyright 2021, American Chemical Society Publications.

As shown in Figure 3a,b [71], a cholesterol tail was conjugated to both the DNAzyme and its substrate at the 5′‐end, enabling their insertion into the membrane of living cells via hydrophobic interactions. The 3′‐end of each strand was labeled with a fluorescent marker, allowing real‐time fluorescence tracking. These modified strands were subsequently incorporated into the membranes of DNAzyme‐linked and substrate‐linked cells, respectively. Through hybridization between the DNAzyme and its substrate, cell–cell assemblies formed. The disassembly of these complexes could be triggered by specific cofactor metal ions that cleave the substrate at the ribonucleotide site [71, 72, 73]. Furthermore, DNAzymes with specificity for different metal ions can be combined to achieve sophisticated regulation of dynamic cellular behaviors. For example, integrating Mg^2+^‐ and Zn^2+^‐specific DNAzymes enables the construction of a dual‐input molecular switch capable of performing AND and OR logic operations [71, 74, 75].

The DNAzyme and its substrate strand exhibit a melting temperature of approximately 60°C, which is significantly higher than the cell culture temperature of 37°C, ensuring stable binding under physiological conditions. In contrast, the cleaved substrate fragments melt at around 20°C, indicating that cleavage induced by metal ions can efficiently occur at 37°C [76]. Using HeLa cells as a model system, the researchers validated the concept by demonstrating that Zn^2+^ pretreatment of the DNAzyme–substrate mixture resulted in catalytic cleavage, as evidenced by gel electrophoresis. Moreover, mixing cells labeled with chain‐1 (FAM‐tagged) and chain‐2 (Cy5‐tagged) in equal proportions led to the fastest assembly rate, and the assembled state persisted for up to 24 h post‐labeling. No significant difference was observed in the assembly and disassembly behaviors between PBS and cell culture conditions. Additional experiments confirmed that DNAzyme catalysis is essential for assembly, as mutations in either the catalytic core or the substrate binding region abolished complex formation (Figure 3c) [71].

For implementing AND logic gates, the authors designed AND chain‐1, which contained a 3′‐end lipid anchor, a Zn^2+^‐specific DNAzyme (DNAzyme‐1), a substrate cleavable by an Mg2+‐specific DNAzyme, and a FAM label. Similarly, AND chain‐2 was equipped with a lipid anchor, an Mg^2+^‐specific DNAzyme (DNAzyme‐2), a substrate cleavable by a Zn^2+^‐specific DNAzyme (substrate‐1), and a Cy5 label. Results showed efficient cell assembly, with disassembly occurring only in the presence of both Zn^2+^ and Mg^2+^, consistent with AND logic behavior (Figure 3d) [71].

The OR logic operation was achieved by reconfiguring the two‐chain system: the positions of substrate‐1 and DNAzyme‐2, as well as DNAzyme‐1 and substrate‐2, were swapped to form OR chain‐1 and OR chain‐2. In this configuration, disassembly could be induced by either Zn^2+^ or Mg^2+^, fulfilling the OR logic function. The disassembly rate was found to be dependent on metal ion concentration (Figure 3e) [71].

### 3.3 DNAzyme‐Based Molecular Machines for Dynamic Inter‐ and Intracelluar Regulation

Combination therapy has emerged as a promising anticancer strategy that integrates immunotherapy with nanoparticle‐based drug delivery systems. This approach aims to enhance intracellular drug concentrations while simultaneously activating the immune system to eliminate cancer cells. Despite its conceptual appeal, this strategy still suffers from insufficient efficacy in meeting clinical demands—particularly in T‐cell‐based therapies, where the absence of effective targeting molecules to bridge T cells and cancer cells limits its application [77, 78, 79].

**FIGURE 4:**
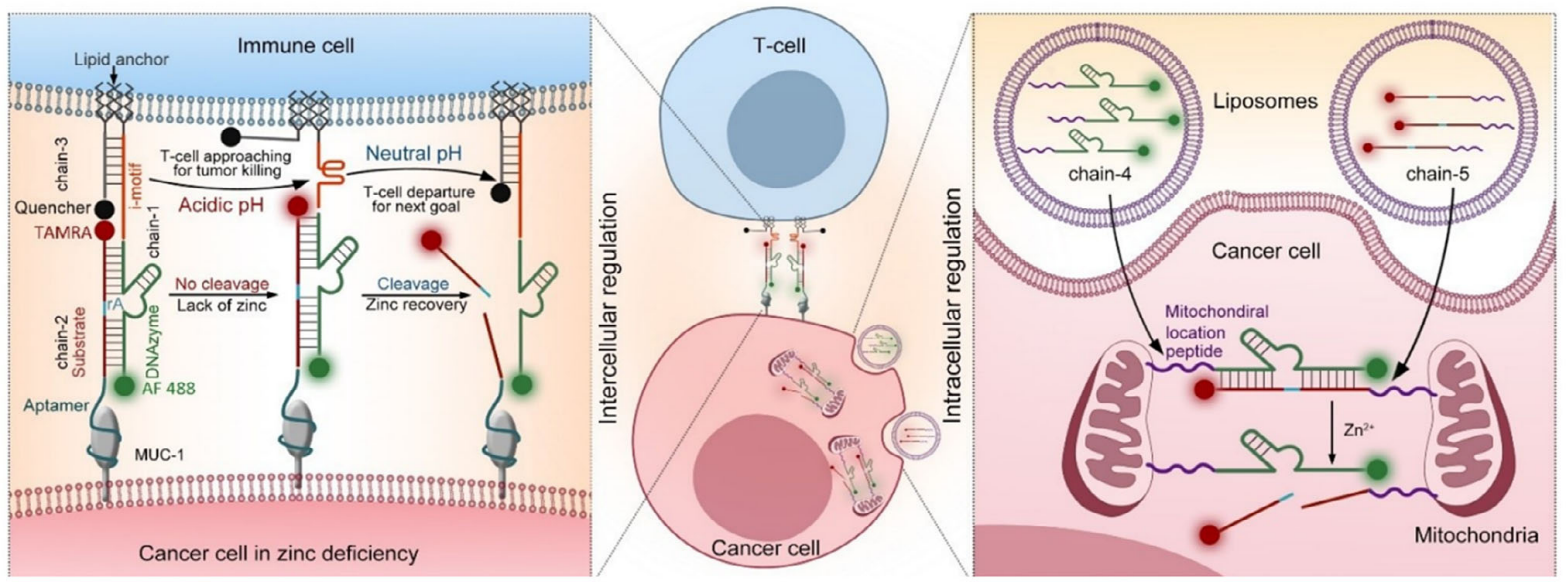
Schematic illustration of a synergistic cancer treatment strategy based on engineered DNAzyme molecular machines. (Left) Multilevel dynamic regulation of T cell‐cancer cell interactions mediated by Chain 1, Chain 2, and Chain 3. Changes in the distance between T cells and cancer cells can be monitored in real time via TAMRA fluorescence signals. (Right) Precise regulation of mitochondrial aggregation within cancer cells achieved using Chain 4 and Chain 5. This phenomenon occurs exclusively in zinc‐deficient cancer cells. Reproduced with permission. [Ref. 70] Copyright 2022, John Wiley & Sons, Inc.

To address this limitation, Yi Lu and colleagues designed a combination therapy strategy based on molecular machines that incorporate engineered DNAzymes and DNA aptamers. This system induces cancer cell apoptosis through extracellular regulation of T‐cell/cancer‐cell interactions and intracellular control of mitochondrial assembly. The team modified a Zn^2+^‐dependent DNAzyme (Chain‐1) with dual lipid tails at the 5′‐end, enabling spontaneous insertion into living cell membranes via hydrophobic interactions. An i‐motif sequence was inserted between the DNAzyme catalytic core and the lipid tail, allowing pH‐sensitive structural changes to modulate intercellular distance. The corresponding substrate chain (Chain‐2) was functionalized at the 5′‐end with an aptamer that specifically binds to MUC‐1 proteins overexpressed on cancer cell surfaces, facilitating targeted binding. The 3′‐ends of Chains 1 and 2 were labeled with Alexa Fluor 488 (AF 488) and carboxytetramethylrhodamine (TAMRA), respectively. These fluorophores remain stable under acidic conditions, making them suitable for real‐time fluorescence tracking within the tumor microenvironment. Furthermore, the distance between cancer cells and T cells was regulated using a complementary i‐motif strand with a 3′‐end lipid anchor (Chain 3), which was embedded into the T‐cell membrane and hybridized with the i‐motif region of Chain 1. The intercellular distance was monitored based on the quenching efficiency between BHQ2 and TAMRA. This DNAzyme‐based molecular machine enables precise regulation of cancer cell‐T cell interactions, sensing and responding to changes in the tumor microenvironment. It thereby facilitates targeted T‐cell/cancer‐cell approximation, cancer cell recognition, and post‐treatment separation (Figure 4) [70].

## 4 DNAzyme‐Based Bioimaging

Bioimaging is a rapidly advancing field that leverages digital technologies, which is still in a phase of rapid development. This technology aims to achieve real‐time dynamic visualization of biological processes by integrating anatomical structural information with various functional data such as magnetic fields, electric fields, mechanical motion, and metabolism. It provides a more comprehensive understanding of human anatomical structure and functional status. As a noninvasive technique, bioimaging not only offers a holistic view of the internal human body but also delivers increasingly rich and detailed morphological and functional information as the technology evolves. It has become an indispensable tool for investigating physiological processes and underlying disease mechanisms. In bioimaging systems, signal transduction is a core component enabling high‐precision detection. DNAzyme‐based signal transduction strategies encompass multiple technical approaches, including fluorescence, electrochemical, bioluminescence, chemiluminescence, and colorimetric methods. These provide highly flexible and specific detection options for disease diagnosis, biomedical research, and environmental monitoring, demonstrating broad application prospects [80, 81, 82, 83].

### 4.1 DNAzyme‐Based Fluorescence

Fluorescence occurs when certain compounds known as fluorophores absorb photons at specific wavelengths, causing their electrons to transition to higher energy levels. When these excited electrons return to their ground state, they emit fluorescence with a longer wavelength and lower energy than the excitation light. This principle has been widely applied in biochemistry and materials science, including fields such as fluorescence microscopy, molecular imaging, and biosensing.

However, several challenges remain in cellular imaging applications: background fluorescence reduces image contrast and detection specificity, while photobleaching limits the effective imaging time. Therefore, careful optimization of imaging conditions and fluorophores is required [84, 85, 86].

**FIGURE 5:**
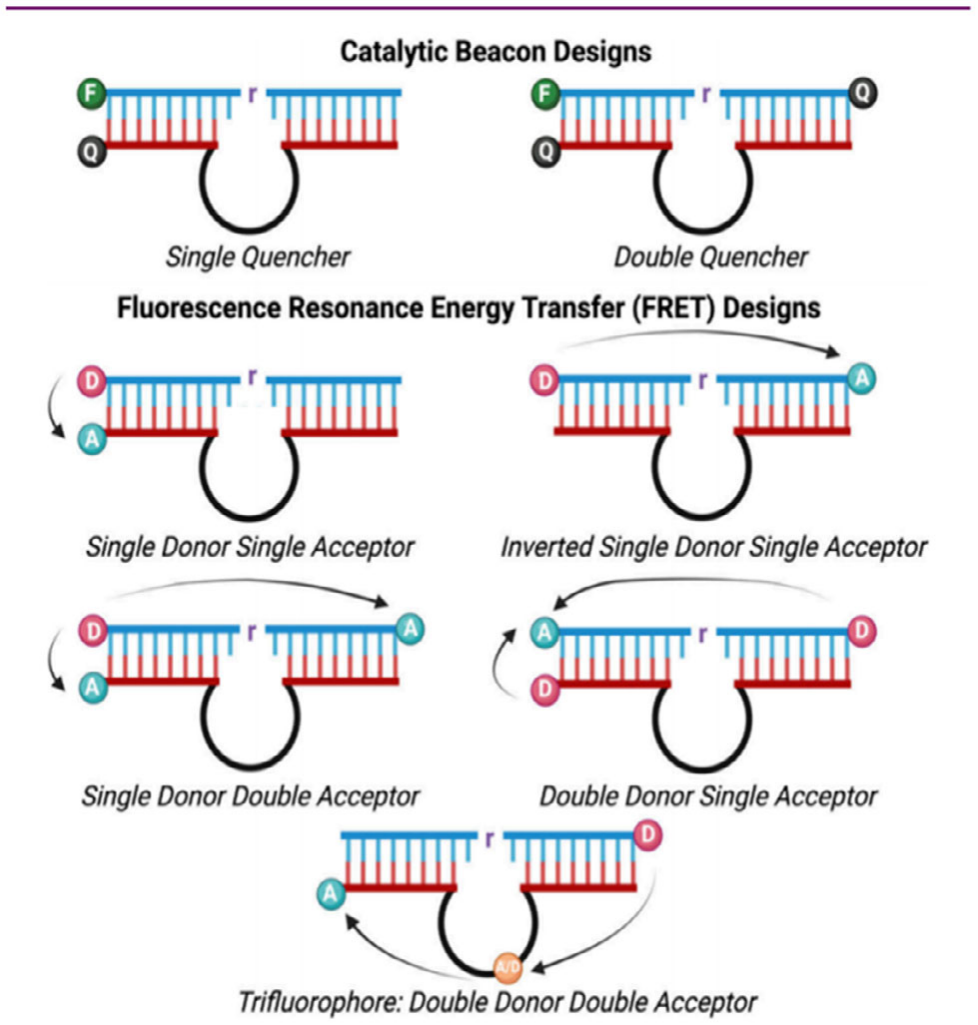
In systems combining small organic fluorophores with DNAzymes, catalytic beacons and FRET represent two core design strategies. Catalytic beacons adopt a hairpin‐structured oligonucleotide probe design that generates a fluorescent signal upon hybridization with a target sequence. This configuration brings the fluorophore and quencher into close spatial proximity, significantly enhancing both sensitivity and specificity for detecting low concentrations of nucleic acid molecules. Reproduced with permission. [Ref. 33] Copyright 2025, American Chemical Society Publications.

DNAzyme‐based fluorescent biosensors represent an emerging hotspot in this field. They detect interactions between analytes through changes in fluorescence intensity. Among them, the catalytic beacon strategy is particularly notable: these crescent‐shaped oligonucleotide probes emit fluorescence upon binding to target nucleic acids. This design brings the fluorophore and quencher into close proximity, enabling highly sensitive and specific detection of low‐concentration nucleic acids. Moreover, their simple structure and ease of modification make them adaptable to a variety of applications [87, 88]. Another key technology is fluorescence resonance energy transfer (FRET), which relies on nonradiative energy transfer between donor and acceptor fluorophores, enabling the detection of molecular interactions at the nanoscale. These two core strategies have significantly advanced the development of fluorescence sensing technology (Figure 5) [33].

### 4.2 Ribosomal RNA (rRNAs) Regulated DNAzyme Sensor

**FIGURE 6:**
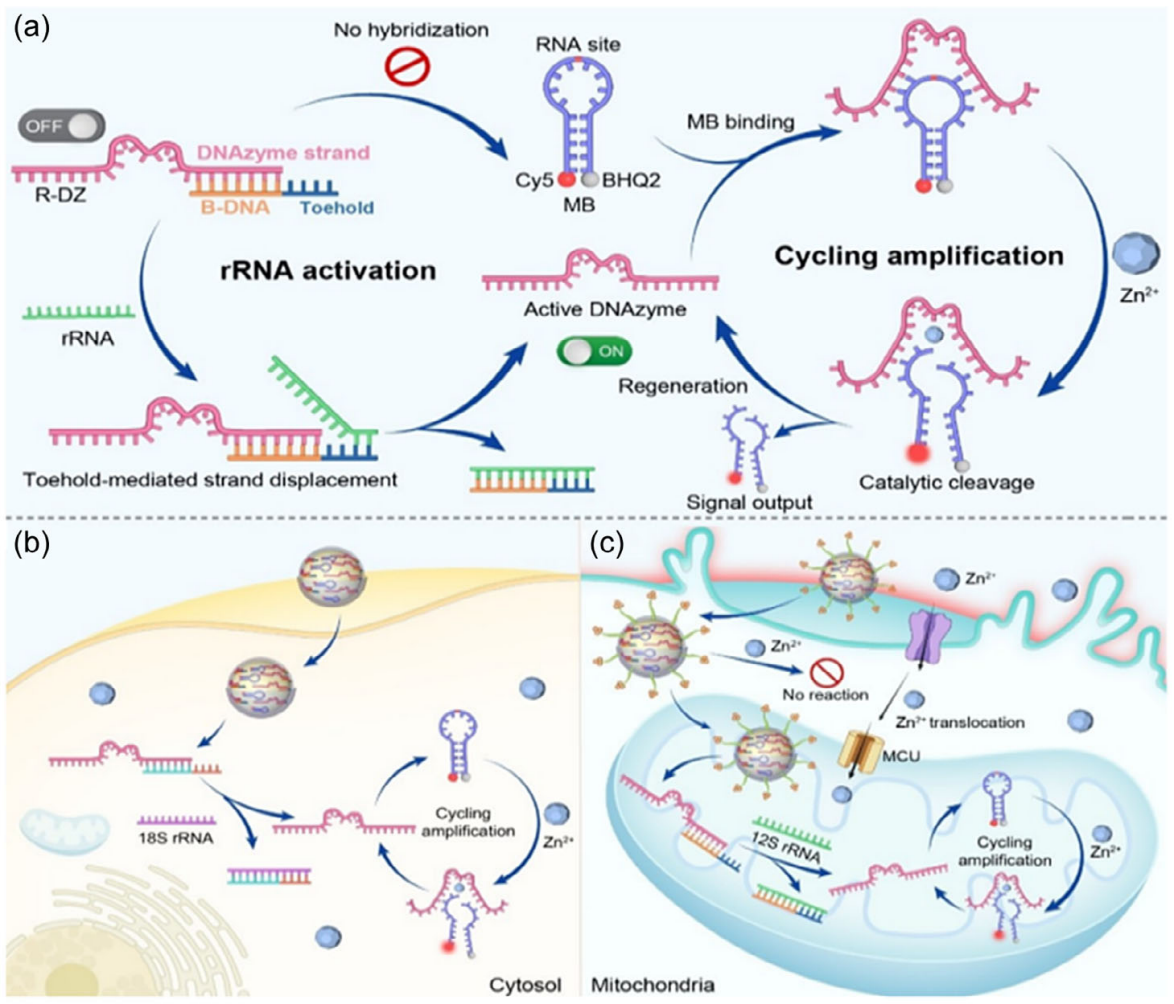
Schematic of subcellular compartment‐specific metal ion imaging technology: Locally activatable DNAzyme sensing strategy based on rRNA‐regulated signal amplification. (a) Working principle of the rRNA‐regulated DNAzyme sensor (R‐DZ/MB). (b) rRNA‐regulated DNAzyme nanosensor for compartment‐specific amplified imaging of Zn^2+^ in the cytosol. (c) rRNA‐regulated DNAzyme nanosensor for compartment‐specific amplified imaging of Zn^2+^in mitochondria. Reproduced with permission. [Ref. 89] Copyright 2025, John Wiley & Sons, Inc.

Despite these significant advantages, most DNAzyme sensors still suffer from inherent limitations. For instance, they remain perpetually active and are prone to interference from metal ions encountered during delivery, leading to nonspecific signal leakage. Therefore, developing stimuli‐responsive DNAzyme sensors has become a key research focus. Consequently, the development of stimuli‐responsive DNAzyme sensors has become a key research focus. The research group led by Mengyuan Li proposed a rRNA‐regulated DNAzyme sensor technology, which enables size‐restricted metal ion imaging in membrane‐less compartments (e.g., cytosol) or specific organelles (e.g., mitochondria) [89]. This strategy utilizes two types of subcellular rRNAs, namely, cytosolic 18S rRNA and mitochondrial 12S rRNA, as endogenous regulatory factors to achieve precise control over subcellular metal ion imaging. The rRNA‐regulated DNAzyme sensor (abbreviated as RDZ/MB) consists of two core modules: first, an rRNA‐activatable DNAzyme sensing unit (R‐DZ), and second, a molecular beacon (MB)‐based signal amplification module. The R‐DZ is engineered from a Zn^2+^‐specific 17E DNAzyme by incorporating a blocker DNA (B‐DNA) with a toehold region. The binding of B‐DNA to the DNAzyme prevents the enzyme strand from hybridizing with the substrate strand embedded within the MB loop, thereby inhibiting the DNAzyme's catalytic activity. The MB module is constructed with a fluorophore/quencher pair (Cy5/BHQ2) at the ends of its extended stem and contains a ribonucleotide cleavage site (rA, adenosine ribonucleotide) within the loop region. This design ensures a low fluorescence background via the Förster resonance energy transfer (FRET) mechanism. Upon introduction of rRNA, B‐DNA forms a more stable duplex with rRNA through toehold‐mediated strand displacement, thereby releasing the active DNAzyme. In the presence of Zn^2+^ ions, the DNAzyme cleaves the substrate strand within the MB, splitting it into two fragments. This cleavage leads to dissociation of the MB stem structure and subsequent fluorescence recovery, enabling metal ion detection. Due to reduced hybridization stability, the DNAzyme is released from the cleaved MB fragments and can participate in subsequent catalytic cycles, achieving signal amplification through enzymatic multiple turnovers (Figure 6) [89].

## 5 Applications of DNAzyme Nanostructures in Cellular Therapy

DNAzyme nanostructures have emerged as a powerful tool in cellular therapy, leveraging their precise gene‐silencing capabilities and programmable responsiveness. Below are key applications and research highlights: Developing theranostic nanosystems that integrate cascaded SERS imaging with gene silencing therapy is a major pursuit in the field of precision cancer diagnosis and treatment [90, 91]. Chen Dong et al. innovatively constructed a dual‐component theranostic system based on AuNPs (AuNP‐Ys/AuNP‐Ds), which achieves specific miRNA recognition through Y‐motif surface modification and constructs a signal amplifier by co‐labeling Raman molecules and double‐stranded DNA linkers. Driven by ATP, the system undergoes miRNA‐triggered conformational transitions, releasing miRNAs for recycling and self‐assembling into AuNP network nanostructures. This enables both enhanced SERS signals for highly sensitive cancer cell detection and specific activation of DNAzymes to catalyze the cleavage of key mRNAs (Survivin/c‐Jun), simultaneously accomplishing diagnosis and dual‐gene silencing therapy. The innovative system has achieved significant breakthroughs in specificity, detection sensitivity, and therapeutic efficacy through its three‐stage cascade reaction of target recognition, SERS imaging, and gene therapy, as demonstrated by tumor mouse models that validated its precise targeting capability and theranostic synergy [92]. This research provides new insights for the development of next‐generation intelligent cancer theranostic platforms.

Breast cancer remains one of the leading causes of death among women worldwide, necessitating breakthrough therapeutic strategies [93, 94]. While photodynamic therapy (PDT) based on miR‐21 imaging shows therapeutic promise, it faces two major bottlenecks: low sensitivity of miR‐21 detection at tumor sites and insufficient PDT efficiency [95, 96]. The Ce6‐DNAzyme@ZIF‐8@PEG nanoparticles (CDZP NPs) developed by Zeping Yang et al. innovatively integrate a dual‐cycle signal amplification system with GPX4‐DNAzyme gene‐editing technology, establishing a tri‐functional diagnostic‐therapeutic system: Firstly, the one‐pot encapsulation technique based on the ZIF‐8 MOF framework enables efficient integration of miR‐21 imaging systems with Ce6‐DNAzyme therapeutic modules. Its acid‐responsive release properties (Zn^2+^/Ce6) precisely target the tumor microenvironment while elevating miR‐21 detection sensitivity to 3.4 pM. Secondly, Zn^2+^‐activated GPX4‐DNAzyme specifically inhibits GPX4 protein expression, effectively blocking Reactive Oxygen Species (ROS) scavenging pathways, thereby significantly enhancing PDT tumor suppression efficacy to 72.3%. Finally, tumor‐bearing mouse models validated the system's precise targeting capability and therapeutic synergy, offering an innovative paradigm for breast cancer precision diagnosis and treatment [97]. This study achieves substantial improvements in diagnostic accuracy and therapeutic efficacy through a multidimensional synergistic approach combining molecular imaging, gene editing, and PDT.

As a DNAzyme specifically targeting c‐Jun mRNA, Dz13 demonstrates remarkable therapeutic efficacy in suppressing squamous cell carcinoma growth [98]. However, conventional DNAzyme therapeutics face significant delivery challenges due to poor cellular membrane permeability and inefficient internalization [99, 100]. To address these limitations, the research team led by Lingxian Meng developed an innovative covalent conjugation strategy, anchoring the Dz13 catalytic sequence to the 5′ terminus of single‐stranded DNA. Through self‐assembly techniques, they engineered a stable tetrahedral DNA nanostructure (TDN‐Dz13). Experimental validation revealed that this nanocarrier system not only enhanced cellular uptake efficiency by over threefold compared to free Dz13 but also fully preserved its gene‐silencing functionality. The system achieves precise degradation of c‐Jun mRNA, resulting in tumor cell proliferation inhibition rates exceeding 85%. This therapeutic‐delivery integrated approach, combining nucleic acid therapeutics with intelligent delivery systems, provides a novel solution to overcome the clinical translation barriers of DNAzyme‐based therapies [101].

## 6 Summary and Future Outlooks

This review systematically explores the diverse functionalities of DNAzyme‐based nanostructures in dynamic cellular regulation and sensing, with a focus on their significant advancements in biosensing, controllable cell–cell interactions, and bioimaging. As catalytic DNA molecules, DNAzymes exhibit remarkable advantages such as high stability, ease of synthesis, and flexible functionalization, making them indispensable tools for metal ion detection, cellular behavior modulation, and precise bioimaging. The integration with nanotechnology has effectively mitigated their inherent limitations, including susceptibility to nuclease degradation and low cellular uptake efficiency, thereby significantly enhancing their practical applicability in complex biological environments.

Key technological advancements include the development of logic‐gated systems for programmable cell assembly and disassembly, rRNA‐activated sensors for subcellular imaging, and DNAzyme‐driven nanodevices (e.g., DNA walkers and molecular tweezers) for signal amplification and intracellular manipulation. These innovations have facilitated the development of novel therapeutic strategies, such as targeted gene silencing and combination therapies, demonstrating broad application prospects in cancer treatment and cellular engineering.

Looking ahead, the evolution of DNAzyme‐based nanotechnology will be driven by the pursuit of greater biological sophistication, functional integration, and translational readiness. Specific research priorities can be outlined across three interconnected frontiers: (1) Toward Intelligent, Conditionally Active Nanodevices. Moving beyond simple stimulus–response, the next generation of nanocarriers must exhibit integrated logic processing akin to biological circuits. Research priorities include: Multi‐input Activation Systems: Designing DNAzyme circuits that require the simultaneous presence of multiple disease‐specific biomarkers (e.g., a specific mRNA and an overexpressed surface protein and a dysregulated metal ion) to trigger activity, achieving unprecedented specificity for diseased cells. Closed‐Loop Feedback Control: Engineering systems where the catalytic output of a DNAzyme (e.g., generation of a therapeutic agent) is dynamically regulated by the changing concentration of its target, enabling self‐titrating, adaptive therapy. Advanced Trigger Mechanisms: Exploiting novel biochemical cues beyond pH and redox, such as disease‐specific enzyme activities (e.g., matrix metalloproteinases), aberrant metabolite concentrations, or even physical cues like local stiffness. (2) Convergence for Multimodal Theranostic Platforms. The true power of DNAzyme nanodevices lies in their integration with complementary technologies. Key opportunities exist at the convergence with: CRISPR‐Based Systems: Combining DNAzymes’ continuous, catalytic RNA cleavage with CRISPR‐Cas's programmability and DNA‐targeting capability for multiplexed gene regulation and editing. Immuno‐Engineering: Coupling DNAzyme‐driven antigen release or immunomodulatory gene silencing with checkpoint inhibitor delivery to create potent in situ cancer vaccines or to remodel the tumor microenvironment. Advanced Imaging Modalities: Moving beyond fluorescence to integrate DNAzymes with photoacoustic imaging (for deep‐tissue penetration), Raman imaging (for multiplexed, background‐free detection), or magnetic particle imaging (for quantitative, whole‐body tracking), thereby correlating therapeutic action with high‐fidelity, real‐time readouts. (3) Addressing Translational Roadblocks. To bridge the gap from promising prototypes to clinical impact, focused efforts are needed on: Scalable and Reproducible Fabrication: Developing robust, cost‐effective manufacturing processes (e.g., microfluidic‐assisted assembly, enzymatic mass production) for complex DNAzyme nanostructures that meet Good Manufacturing Practice (GMP) standards. Comprehensive In Vivo Pharmacokinetics/Pharmacodynamics (PK/PD) Profiling: Systematically evaluating the fate, durability of effect, and potential immunogenicity of DNAzyme nanodevices in large animal models, which is critical for dosage and regimen design. Standardization and Benchmarking: Establishing universally accepted metrics and model systems to objectively compare the performance of different DNAzyme designs, accelerating the identification of lead candidates. By addressing these focused priorities—intelligence through molecular computation, power through technological convergence, and reliability through engineering rigor—DNAzyme‐based nanotechnologies are poised to transition from sophisticated laboratory tools to transformative clinical solutions.

## Supporting Information

Additional supporting information can be found online in the Supporting Information section.

## Author Contributions

**Ruocan Qian**: conceptualization (lead), funding acquisition (lead), supervision (lead), writing – review & editing (lead). **Haoyu Fan**: validation (lead), writing – original draft (equal), writing – review & editing (equal). **Xichen Du**: (writing – original draft: equal; writing – review & editing: equal).

## Funding

This study was supported by Fundamental Research Funds for Central Universities of the Central South University (222201717003), Science and Technology Innovation Plan Of Shanghai Science and Technology Commission (22ZR1416800), Innovative Research Group Project of the National Natural Science Foundation of China (21977031).

## Conflicts of Interest

The authors declare no conflicts of interest.

## Supporting information

Supplementary Material

## Data Availability

The data that support the findings of this study are available in the supplementary material of this article.
